# Indigenous Proximal Femoral Nails and Their Novel Complications

**DOI:** 10.7759/cureus.16729

**Published:** 2021-07-29

**Authors:** Naveen Kumar, Pulak Vatsya, Amit K Salaria

**Affiliations:** 1 Orthopaedics, Post Graduate Institute of Medical Education & Research, Chandigarh, IND; 2 Orthopaedics, All India Institute of Medical Sciences, New Delhi, IND

**Keywords:** trochanter fractures, pfna, geriatric, complications, indiginous implants

## Abstract

Pertrochanteric fractures are one of the commonest fractures in the geriatric age group. Management of these fractures has been revolutionized by the use of proximal femoral nails (PFN), with a screw or a helical blade for fixation in the femoral head. Multiple complications like Z-effect, screw cut out, head penetration, varus collapse, and so on are known with poor technique in proximal femoral nails antirotation (PFNA). We present a case where an indigenous implant presented to us a novel problem of helical blade breakage inside the bone in situ. Removal of this blade needed an open approach, extending the surgical time as well as blood loss. This led to poorer outcomes and the intraoperative struggle for the surgeon. We feel that all surgeons, especially when using indigenous implants, should be aware of such complications and thoroughly check the helical blade and its collapsing mechanism before inserting this in the bone.

## Introduction

Pertrochanteric fractures are one of the commonest fractures, especially in the geriatric age group. Multiple fixation devices, extramedullary or intramedullary are used for fixation with no clear advantage of one over the other, and with both having their own advantages over the other as demonstrated by Sun et al. in one of their studies [1]. The past decade has seen proximal femoral nail antirotation (PFNA) becoming the go-to implant for surgeons to fix these fractures due to the shorter duration of surgery, minimal blood loss, good fixation even in unstable fractures, and early mobilization of patients, thus avoiding the complications arising from recumbency as elucidated by Makridis et al. [2]. With the widespread use of PFNA, complications like head perforation by the helical blade, cut-out of the blade, varus collapse, fracture near the tip of the, and so on have rapidly raised in number as shown by Audig et al. in their data. [3]. In a developing world, where multiple indigenous manufacturers are making implants, some standardized and some not, operating surgeons are exposed to newer intra-operative struggles with the implant leading to prolonged surgical duration, increased blood loss, larger incisions as well as poorer outcomes. Only a single article enlists intra-operative inability to lock the blade and another case where mismatch of jig and the blade was reported, causing the intra-operative struggle shown by Siu et al. [4]. We report a case, where the helical blade of the implant broke while trying to remove the blade from the bone due to a size mismatch. This complicated the procedure further and needed conversion to an open procedure, thus defeating the basic purpose of choosing PFNA over dynamic hip screw (DHS).

## Case presentation

An 85-year-old female, with a history of falls at home, presented to us with pain in the right hip. On radiographs, a communited unstable intertrochanteric fracture was noted. The patient was planned for PFNA on a traction table.

**Figure 1:**
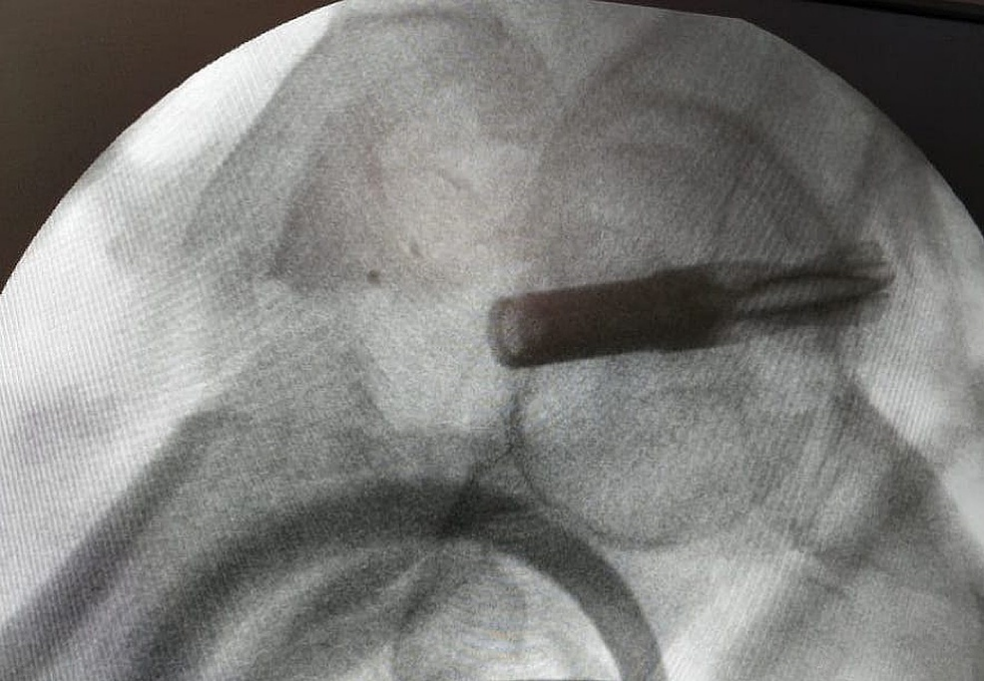
Broken helical blade pushed into the joint

The patient was positioned on a fracture table, and a closed reduction is done. Once a satisfactory reduction was achieved, the incision for standard PFNA was made. Once the nail was passed, the lateral incision for the blade was made. The bone was drilled with an appropriate drill, and keeping the tip-apex distance to be <25mm, a screw of size 90cm was chosen. On passing the blade, it was seen on the c-arm that the blade was greater than expected and would lead to joint penetration and hence, needed to be revised to a smaller size. For this, the blade was removed and a new blade of size 80cm was taken and driven along the same path. The surgeon felt resistance while reaching the end of the blade, and on seeing in the c-arm, a part of the previous blade was found to be inside the head and had been pushed further across the joint, due to the force by the new blade (Figure 1).

**Figure 2:**
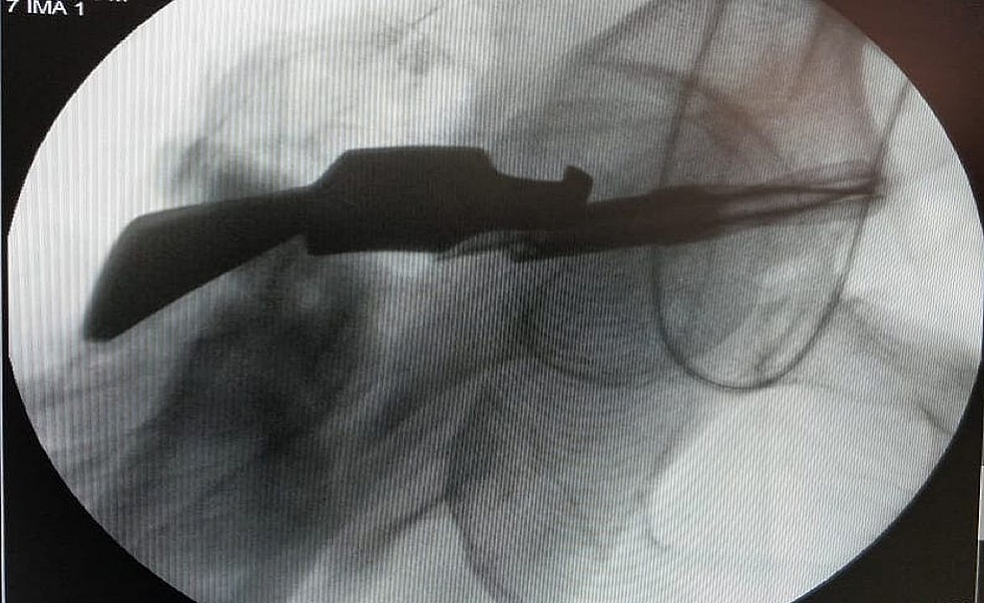
Broken blade being removed with help of a retractor through an open approach and fracture distraction

**Figure 3:**
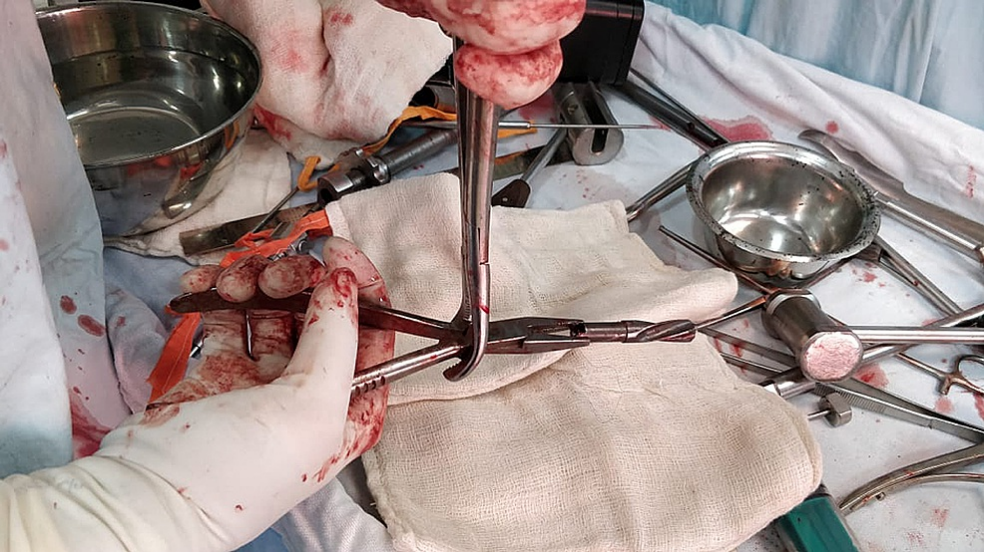
Bone nibbler and multiple other instruments used to pull out the broken blade

**Figure 4:**
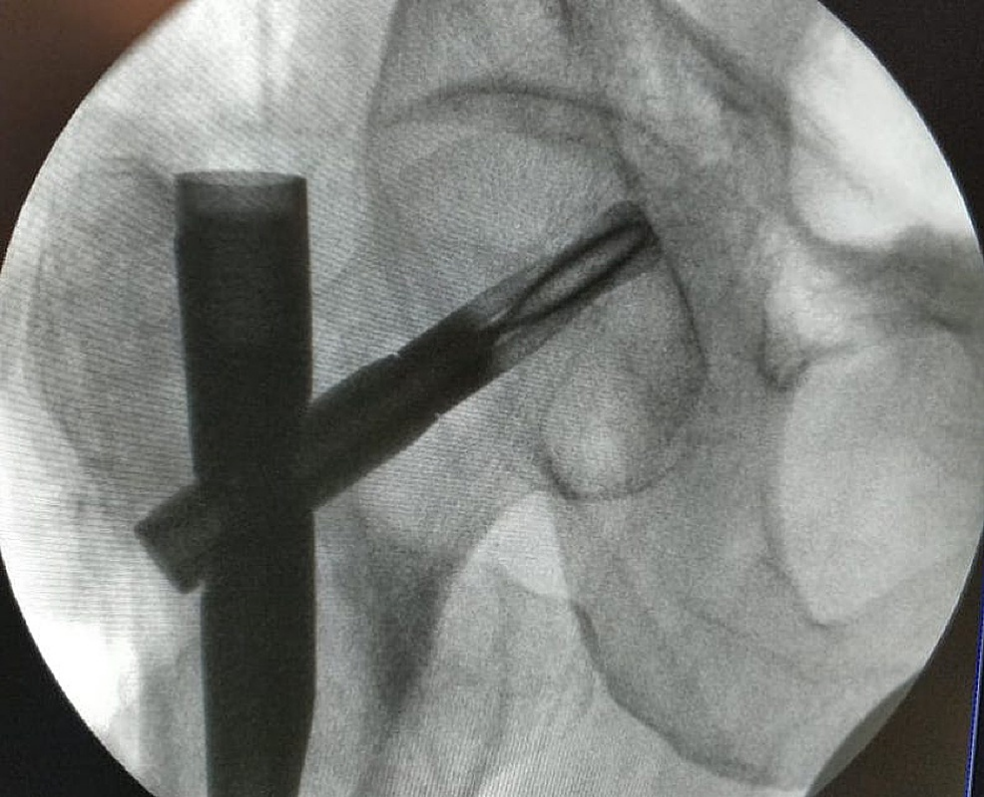
Final radiograph with nail and the new blade in situ

The surgeon checked the part that had been removed earlier and found out that it was only the sleeve that had been removed, and the helical blade had broken and was left in situ. The surgeon decided to use a Watson Jones approach to open the fracture and expose the head and remove the helical blade with a nibbler (Figure 2, 3). Once removed, the fracture was reduced, and the nail was reinserted and a new blade of size 80cm was put in (Figure 4). The wound was closed in layers with a drain in situ, and the patient was kept non-weight bearing for 6 weeks.

Post-operative course

The patient was kept non-weight bearing for 6-8 weeks till there were signs of new bone formation. This was warranted by the operating surgeon due to the highly osteoporotic nature of the bone, and fear of screw cut out on early weight-bearing, even though the tip-apex distance was appropriate. The patient was subsequently started on toe-touch weight-bearing with a walker and slowly converted to partial and then full weight-bearing. The patient continued to have pain and difficulty in weight-bearing even at 4 months postoperatively. The patient expired at 8 months from surgery due to natural causes.

## Discussion

PFNA has become the workhorse for management of pertrochanteric fractures especially in the elderly due to multiple advantages over the DHS including the shorter duration, smaller incisions, minimal blood loss, early mobilization, and an easier technique with which more surgeons are familiar since it is similar to the technique used for the interlocking nail of the femur. Post-operative complications like shortening, malunion, nonunion, malposition causing cut-out, Z-effect, lateral cortex blow out and varus collapse have been known and technique modifications have been devised to avoid these as shown by multiple authors like Sadic et al. [5]. However, intraoperative complications are faced commonly but are not frequently reported. The literature review showed just a single report of two cases, in which the author had elucidated intraoperative complications, one being from an inability to lock the helical blade, and another case when there was a mismatch of the jig, due to which the blade could not be passed through the nail as reported by Siu et al. [4]. Also, joint disruption by the blade further would aggravate the process of arthritis in this age group and can be a probable cause of painful weight bearing even after 4 months of the surgery. This disruption of the joint should be avoided at all costs. 

Our case was a standard pertrochanteric fracture, which was planned to be managed with PFNA. Breaking of the helical blade locking sleeve intraoperatively was not expected by the surgical team and was initially missed. Later, this was found on c-arm imaging and lead to the opening of the joint and fracture site, thus belittling all the benefits of closed PFNA. PFNA instrumentation sets have a separate instrument for the head screw removal, in case the surgeon wishes to change the size or is not satisfied with the reduction. Even though we used the designated screwdriver and instrumentation for removal, the locking sleeve of the blade broke and was removed instead of the full blade. This further complicated the procedure. As young surgeons, such a complication of partial blade removal, with some part of it left in the blade tract is almost unknown of and thus the operating surgeon was unwary of this complication. The surgeon only realized this complication when he felt an obstruction on reinserting a new screw in the same tract. This case not only highlights the need to thoroughly check the blade and its collapsing mechanism to be working efficiently before insertion, but also emphasizes the need to choose the right blade size in one go. A lot of surgeons choose a 10-15cm smaller blade than measured, keeping the magnification factor in mind. We feel, with standard devices, a 5-10mm smaller blade than measured should be enough to allow for collapse. TAD>25mm shall again lead to a higher risk of failure. Thus, the right measurement and checking of blade collapse mechanism before insertion should be a routine task similar to checking of the jig and its proximal locking hole sleeve mechanism before insertion of nail. This applies more to non-standardized indigenous nails which are rampantly used in the developing world. 

## Conclusions

Nailing of pertrochanteric fractures in the elderly population should be attempted with all caution. Adequate planning, right implant, and adequate reduction are a must. When using an indigenous implant, an adequate sized blade, avoiding revision of blade or screw as well as checking the blade collapse assembly before insertion is important as these can be potential pitfalls and lead to poorer outcomes.
